# Supplementary material for: Dynamic transcriptomic profiles of zebrafish gills in response to zinc supplementation
Source: BMC Genomics. 2010 Oct 11;11:553. doi: 10.1186/1471-2164-11-553 (PMC3091702; doi:10.1186/1471-2164-11-553)
Supplement: Additional file 2 — Interactive Direct Interaction Network representing the molecular interactions between zinc, copper, iron, calcium and proteins encoded by transcripts changed by zinc supplementation. Mini web-site containing index.html and hyperlinked pages in subdirectory describing a Direct Interaction Network automatically generated based on curated interactions contained within the proprietary PathwayArchitect database. Ovals represent proteins and the circles symbolize metal ions. Objects are coloured by their abundance in zebrafish at the time-point they were significantly different from the control is a scale from -4 fold (dark green) to +4 fold (dark red). Where significant differences were found at more than one time-point, the colour overlay shows expression at the first instance. Dark blue squares denote 'binding', and light blue squares 'expression'; green squares stand for 'regulation', green diamonds for 'metabolism', and green circles for 'promoter binding'. Arrow heads indicate directionality of the interaction where annotated. All nodes and edges can be further interrogated by selecting the relative area of the image. [file 1471-2164-11-553-S2.zip › PathwayArchitect Zn xs DIN/149851.html]

# PROTEIN: CXCL10

|  |  |
| --- | --- |
| Name | CXCL10 |
| Type | PROTEIN |
| Description | chemokine (C-X-C motif) ligand 10 |
| Note | This gene encodes the interferon (gamma)-induced protein of 10kDa, a chemokine of the CXC subfamily that is one of the ligands for the receptor CXCR3. The binding of this protein to CXCR3 causes pleiotropic effects, including stimulation of monocytes, natural killer and T-cell migration, and modulation of adhesion molecule expression. |
| Alias | gamma IP10 |
|  | Inp10 |
|  | 10 kDa interferon- gamma-induced protein |
|  | Mob1 |
|  | Scyb10 |
|  | IP10 |
|  | small inducible cytokine B subfamily (Cys-X-Cys), member 10 |
|  | interferon-inducible cytokine IP-10 |
|  | Cxcl10 |
|  | IFI10 |
|  | protein 10 from interferon (gamma)-induced cell line |
|  | interferon activated gene 10 |
|  | crg-2 |
|  | mob-1 |
|  | Interferon inducible protein 10 |
|  | small inducible cytokine B subfamily (Cys-X-Cys) member 10 |
|  | CXCL10 |
|  | small inducible cytokine subfamily B (Cys-X-Cys), member 10 |
|  | MOB-1 protein |
|  | CRG-2 |
|  | INP10 |
|  | gIP-10 |
|  | C7 |
|  | small inducible cytokine B10 |
|  | SCYB10 |
|  | IP-10 |
|  | Gamma-IP10 |
|  | Ifi10 |


---

|  |  |
| --- | --- |
| GO Component | extracellular space |
|  | extracellular region |
|  | soluble fraction |


---

|  |  |
| --- | --- |
| GO ID | GO:0005615 |
|  | GO:0030335 |
|  | GO:0007600 |
|  | GO:0005489 |
|  | GO:0006118 |
|  | GO:0007166 |
|  | GO:0005576 |
|  | GO:0006935 |
|  | GO:0008009 |
|  | GO:0007165 |
|  | GO:0005125 |
|  | GO:0007267 |
|  | GO:0007517 |
|  | GO:0005625 |
|  | GO:0006954 |
|  | GO:0008015 |
|  | GO:0006928 |
|  | GO:0009306 |
|  | GO:0008603 |
|  | GO:0006955 |
|  | GO:0008284 |


---

|  |  |
| --- | --- |
| MIM | MIM:147310 |


---

|  |  |
| --- | --- |
| Connectivity | 542 |


---

|  |  |
| --- | --- |
| Entrez ID | 3627 |
|  | 15945 |
|  | 245920 |


---

|  |  |
| --- | --- |
| Agilent ID | A\_24\_P303091 |
|  | A\_44\_P1039128 |
|  | A\_23\_P124884 |
|  | A\_51\_P432641 |
|  | A\_42\_P597580 |
|  | A\_53\_P144646 |
|  | A\_53\_P131553 |
|  | A\_14\_P125671 |
|  | A\_53\_P166629 |


---

|  |  |
| --- | --- |
| Cellular Localization | Extracellular region |
|  | Cell |


---

|  |  |
| --- | --- |
| DbXref | KEGG pathway##04620##Toll-like receptor signaling pathway##http://www.genome.jp/dbget-bin/show\_pathway?mmu04620+15945 |
|  | KEGG pathway##04060##Cytokine-cytokine receptor interaction##http://www.genome.jp/dbget-bin/show\_pathway?mmu04060+15945 |
|  | KEGG pathway##04060##Cytokine-cytokine receptor interaction##http://www.genome.jp/dbget-bin/show\_pathway?hsa04060+3627 |
|  | KEGG pathway##04620##Toll-like receptor signaling pathway##http://www.genome.jp/dbget-bin/show\_pathway?rno04620+245920 |
|  | KEGG pathway##04060##Cytokine-cytokine receptor interaction##http://www.genome.jp/dbget-bin/show\_pathway?rno04060+245920 |
|  | KEGG pathway##04620##Toll-like receptor signaling pathway##http://www.genome.jp/dbget-bin/show\_pathway?hsa04620+3627 |


---

|  |  |
| --- | --- |
| Pathway | Zn xs inventory |
|  | Zn xs DIN |


---

|  |  |
| --- | --- |
| GO Process | inflammatory response |
|  | protein secretion |
|  | immune response |
|  | cell surface receptor linked signal transduction |
|  | cell-cell signaling |
|  | chemotaxis |
|  | signal transduction |
|  | positive regulation of cell migration |
|  | electron transport |
|  | cell motility |
|  | positive regulation of cell proliferation |
|  | sensory perception |
|  | muscle development |
|  | circulation |


---

|  |  |
| --- | --- |
| UniGene | Hs.413924 |
|  | Rn.10584 |
|  | Mm.877 |


---

|  |  |
| --- | --- |
| Affymetrix Probeset ID | 1387969\_at |
|  | 1418930\_at |
|  | 204533\_at |
|  | 431\_at |
|  | 93858\_at |
|  | g4504700\_3p\_at |
|  | m33266\_s\_at |
|  | U17035\_s\_at |
|  | X02530\_at |
|  | RC\_AA152305\_s\_at |
|  | TC32016\_at |


---

|  |  |
| --- | --- |
| GO Function | cytokine activity |
|  | cAMP-dependent protein kinase regulator activity |
|  | electron transporter activity |
|  | chemokine activity |


---

|  |  |
| --- | --- |
| Nucleotide | U22520 |
|  | AK157130 |
|  | AK151534 |
|  | AK146144 |
|  | AJ243095 |
|  | AK152568 |
|  | U17035 |
|  | AK172540 |
|  | NM\_139089 |
|  | AK153864 |
|  | BC058444 |
|  | AK152234 |
|  | M86830 |
|  | AK152924 |
|  | X02530 |
|  | AK157589 |
|  | L07417 |
|  | BC010954 |
|  | AK157139 |
|  | AK157678 |
|  | M86829 |
|  | AK144279 |
|  | NM\_001565 |
|  | BC057150 |
|  | AF227743 |
|  | AK150765 |
|  | AK153181 |
|  | AK151415 |
|  | BC030067 |
|  | AK151248 |
|  | NM\_021274 |
|  | AK152838 |
|  | AK152814 |
|  | AK156907 |
|  | AK150987 |
|  | AK150380 |
|  | M33266 |
|  | AK152967 |
|  | AK151210 |


---

|  |  |
| --- | --- |
| Protein | BAE30238 |
|  | BAE31320 |
|  | BAE29832 |
|  | NP\_001556 |
|  | BAE30481 |
|  | AAA75249 |
|  | AAB60485 |
|  | NP\_620789 |
|  | P02778 |
|  | BAE30380 |
|  | BAE33973 |
|  | AAC52811 |
|  | AAA02968 |
|  | CAA26370 |
|  | BAE43058 |
|  | P48973 |
|  | BAE26931 |
|  | NP\_067249 |
|  | AAH58444 |
|  | AAG10400 |
|  | BAE30205 |
|  | BAE34130 |
|  | AAH10954 |
|  | BAE34149 |
|  | BAE33975 |
|  | AAA21489 |
|  | BAE31783 |
|  | BAE31517 |
|  | P17515 |
|  | BAE31599 |
|  | BAE25810 |
|  | BAE29511 |
|  | AAH30067 |


---

|  |  |
| --- | --- |
| Organism | Mammal |


---

|  |  |
| --- | --- |
| Location | 5 53.0 cM (Mus musculus) |
|  | chromosome 4, 4q21 (Homo sapiens) |
|  | chromosome 14, 14p22 (Rattus norvegicus) |
|  | chromosome 5, 5 53.0 cM, 5 E2 (Mus musculus) |


---

|  |  |
| --- | --- |
